# Heterogeneous activation of the TGFβ pathway in glioblastomas identified by gene expression-based classification using TGFβ-responsive genes

**DOI:** 10.1186/1479-5876-7-12

**Published:** 2009-02-03

**Authors:** Xie L Xu, Ann M Kapoun

**Affiliations:** 1 Biomarker R&D, Scios Inc, Fremont, California, USA; 2 Current address: Experimental Medicine, Johnson & Johnson Pharmaceutical Research and Development, San Diego, California, USA; 3 Current address: Department of Translational Medicine, OncoMed Pharmaceuticals Inc, Redwood City, California, USA

## Abstract

**Background:**

TGFβ has emerged as an attractive target for the therapeutic intervention of glioblastomas. Aberrant TGFβ overproduction in glioblastoma and other high-grade gliomas has been reported, however, to date, none of these reports has systematically examined the components of TGFβ signaling to gain a comprehensive view of TGFβ activation in large cohorts of human glioma patients.

**Methods:**

TGFβ activation in mammalian cells leads to a transcriptional program that typically affects 5–10% of the genes in the genome. To systematically examine the status of TGFβ activation in high-grade glial tumors, we compiled a gene set of transcriptional response to TGFβ stimulation from tissue culture and *in vivo *animal studies. These genes were used to examine the status of TGFβ activation in high-grade gliomas including a large cohort of glioblastomas. Unsupervised and supervised classification analysis was performed in two independent, publicly available glioma microarray datasets.

**Results:**

Unsupervised and supervised classification using the TGFβ-responsive gene list in two independent glial tumor gene expression data sets revealed various levels of TGFβ activation in these tumors. Among glioblastomas, one of the most devastating human cancers, two subgroups were identified that showed distinct TGFβ activation patterns as measured from transcriptional responses. Approximately 62% of glioblastoma samples analyzed showed strong TGFβ activation, while the rest showed a weak TGFβ transcriptional response.

**Conclusion:**

Our findings suggest heterogeneous TGFβ activation in glioblastomas, which may cause potential differences in responses to anti-TGFβ therapies in these two distinct subgroups of glioblastomas patients.

## Background

Glial tumors are the most common primary brain malignancies in adults. In the United States, they result in an estimated 13,000 deaths every year [1]. The most aggressive form, glioblastoma (WHO Grade IV), also known as glioblastoma multiforme, is one of the most deadly human malignancies. Glioblastoma patients have a median survival time of less than 12 months despite the standard treatment of surgery, radiotherapy and nitrosourea-based chemotherapy [2]. Significant morbidity and mortality comes from local invasion of the tumor preventing complete surgical resection. Glioblastoma may develop from a diffuse astrocytoma or an anaplastic astrocytoma (secondary glioblastoma), but more commonly presents *de novo *without evidence of a less malignant precursor (primary glioblastoma). Genetically, amplification of the epidermal growth factor receptor (EGFR) locus is found in approximately 40% of primary glioblastomas but is rarely found in secondary glioblastomas; mutations of the tumor suppressor gene *phosphatase and tensin homolog deleted on chromosome 10 *(*PTEN*) are observed in 45% of primary glioblastomas and are seen more frequently in primary glioblastomas than in secondary glioblastomas [3]. Loss of heterozygosity (LOH) of chromosome 10 and loss of an entire copy of chromosome 10, which harbors the *PTEN *gene, are the most frequently observed chromosomal alterations. The aberrant EGFR expression and the mutation of *PTEN *leads to abnormal activation of phosphoinositide-3-kinase (PI3K)/v-akt murine thymoma viral oncogene homolog (AKT) pathway, which provides necessary signals for tumor cell growth, survival and migration [4]. The importance of activation of EGFR-PI3K/PTEN pathway in the pathogenesis of glioblastoma has been confirmed in the subgroup of patients who showed clinical responses to EGFR kinase inhibitors [5,6].

The transforming growth factor-β (TGFβ)-mediated pathway has also been shown to play critical roles in glial tumors. The high-grade malignant gliomas express TGFβ ligands and receptors, which are not expressed in normal brain, gliosis, or low-grade astrocytomas [7-10]. The immunosuppressive cytokine, TGFβ, secreted by the tumor cells interferes with the host antitumor immune response therefore allowing the tumor to escape immunosurveilance [11]. Furthermore, TGFβ may act directly as a tumor progression factor. The growth-inhibition function on normal epithelial cells has been lost in many tumor-derived cell lines [12]. The ability of TGFβ to enhance cell migration promotes tumor growth and invasion in advanced epithelial tumors [13-15].

TGFβ ligands are secreted in latent forms and are activated through cleavage of the carboxyl-terminal latency-associated peptide. Activated TGFβ ligands bind to specific cell surface receptors to form an activated heterodimeric serine/threonine kinase receptor complex. The constitutively active type II receptor phosphorylates and activates the type I receptor upon binding of the activated ligands, which then initiates the intracellular signaling cascade involving the SMAD, a family of proteins similar to the gene products of the *Drosophila *gene "mothers against decapentaplegic" (*Mad*) and the *C. elegans *gene *Sma*. SMAD2 and SMAD3 specifically mediate the signals induced by TGFβ. Phosphorylated SMAD2/3 are released from the receptor complex and bind to SMAD4. The SMAD2(3)/SMAD4 complex is translocated into the nucleus and regulates the transcription of specific target genes. TGFβ may act via the SMAD pathway to either promote or inhibit the transcription of specific genes [16]. The transcriptional profiles induced upon TGFβ stimulation have been examined using microarray technology [17-24]. Diversified yet overlapping transcriptional responses are generated by TGFβ stimulation in different tissues in different species. In general, the expressions of 5–10% genes in the genome are affected upon TGFβ stimulation.

Large-scale microarray analysis has been used in gliomas to identify gene signatures that have the power to predict survival and subclasses of gliomas that represent distinct prognostic groups [25-27]. Gene expression-based classification of malignant gliomas was shown to correlate better with survival than histological classification [28]. In this current investigation, we analyzed the transcriptional responses generated upon TGFβ stimulation from multiple studies. We then used this gene signature to examine the activation status of TGFβ in high-grade gliomas using published microarray data.

## Methods

### Glioma microarray datasets

Two glioblastoma microarray datasets were used in this study: Freije *et al *[25] and Nutt *et al *[28]. The Freije study included 85 tumor samples (dChip133ABGliomasGrdIII_IV.xls) and used the affymetrix U133A and U133B gene chips, which contain more than 45,000 probesets. Consistent with the original publication, the dCHIP [29] normalized expression values were used in the analysis. The quality of the data was examined by scatter plots and correlation coefficients were calculated among all samples. 5 tumors (GBM 1469, GBM 1544, GBM 2015, GBM 749, GBM 839) were excluded from further analysis due to large artifacts on the scatter plots and low correlation coefficients with the rest of the samples. Between the two replicates of tumor # 975 (OLIGO III 975 and OLIGO III 975.1), OLIGO III 975 was included here, since it showed better quality as assessed from the scatter plot. The average of the two replicates (OLIGO III 744, OLIGO III 744.1) was used for the same reason. A total of 78 tumors from this dataset were used in the subsequent analysis. The second, independent dataset from Nutt *et al *[28] included 50 tumors and was generated on the Affymatrix U95A platform. The files with .cel format were downloaded from  and normalized with GC-RMA in Splus 6.2 (Insightful) with the S+ArrayAnalyzer module (2.0). Pearson's correlation coefficients were calculated among all tumors and 4 tumors (Brain_NG_13, Brain_CG_1, Brain_NG_11, Brain_CG_10) were excluded from further analysis due to low correlation coefficients with the rest of samples. A total of 46 samples from this dataset were used in the following analysis.

### Data analysis

**Figure 1:**
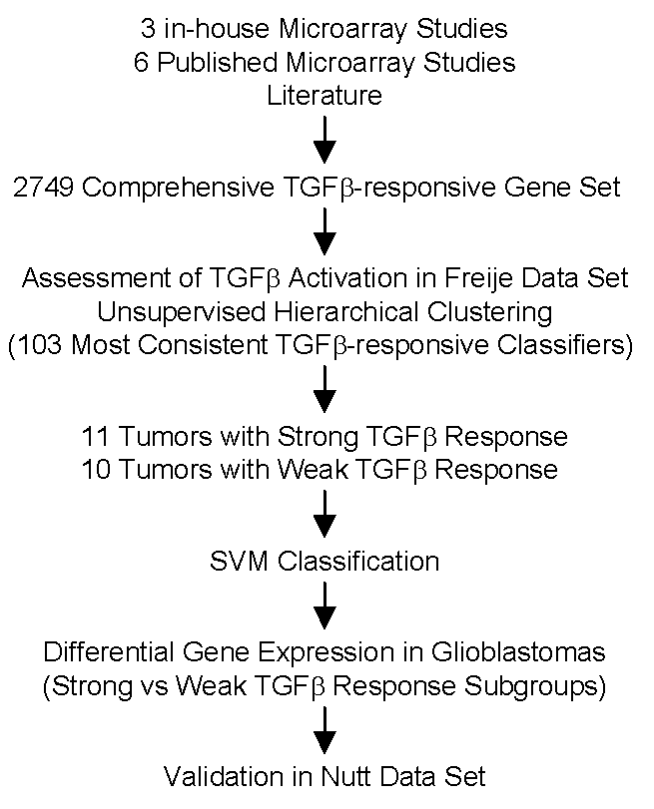
**Outline of data analysis steps**.

ANOVA, t-test, Pearson's correlation coefficient calculations, Support Vector Machine (SVM) classification, and survival analysis were computed using MATLAB 7.1 software (MathWorks, Natick, MA). The hierarchical clustering was performed in Spotfire DecisionSite 8.1 for Functional Genomics (Spotfire, Somerville, MA). The overall outline of the analysis steps is summarized in Figure 1.

### TGFβ-Responsive gene list

The comprehensive TGFβ-responsive gene set was compiled from 3 in-house microarray studies, 6 published microarray studies [19-24], and an in-house curation of >100 publications on TGFβ regulated genes. The 3 in-house microarray studies include: human lung fibroblast +/- TGFβ [17], human glioblastoma cell line LN308 +/-TGFβ (unpublished data), and human pancreatic cancer cell line Panc1 +/- TGFβ [30]. For the published microarray studies, the whole datasets were not always available, however, the differentially expressed gene list based on the authors' criteria was normally presented in the publications. The following strategy was utilized to summarize the results from different studies and publications. For each of the microarray studies, if a gene was identified by the original authors using their criteria as differentially expressed after TGFβ stimulation at any of the time points in the original publication, it contributed one count to this gene. If the gene was one of the in-house curated TGFβ regulated genes, it also contributed one count. For in-house microarray studies where the whole datasets were available, a differentially expressed gene was defined as genes with at least 1.8 fold change in response to TGFβ treatment. If the study was done in mouse models, the human orthologs were identified for the mouse genes through the ortholog map from Mouse Genome Informatics . The counts were then summed across all studies for each gene (Additional file 1: Counts of Studies). The direction of changes after TGFβ treatment was also summarized in the following fashion: upregulation of gene expressions upon TGFβ stimulation contributed positive counts, while downregulation of gene expressions after TGFβ treatment contributed negative counts. The signed counts were then summed across all microarray studies. If one gene is upregulated by TGFβ in one study but downregulated by TGFβ in another study, the direction counts will cancel each other during summarization therefore the total direction counts will be fewer than the total counts of the studies (Additional file 1: Directions). Since the direction of changes in TGFβ regulated genes curated from literature were not readily available in our database, they were not included in the directional counts.

## Results

### Identification of TGFβ-Responsive gene set

To investigate potential TGFβ activation among glial tumors, we first identified a gene set that was responsive to TGFβ stimulation using in-house and public microarray data. Based upon several large-scale gene expression profiling experiments, TGFβ is expected to generate transcriptional responses that would impact 5–10% of the genome in any given tissues and the transcription profiles upon TGFβ stimulation would be quite diversified in different tissues and species [17,19-24]. The transcriptional responses generated by chronic TGFβ stimulation on tumor tissues would also be different from acute TGFβ stimulation on normal tissues and cell lines. With the variability among microarray experiments, the transcriptional profile from a single experiment is not sufficient to identify TGFβ-responsive genes in glioma tumors. We examined the genes differentially expressed upon TGFβ treatment in multiple large-scale gene expression profiling studies from both the majority of the published literature at the time this study was conducted, and data from in-house microarray experiments; these datasets included multiple tissue types in both human and animal models. Together with curating >100 publications on TGFβ-regulated genes, we compiled a comprehensive TGFβ-responsive gene set using the strategy described above. A total of 2749 unique human genes were identified as responsive to TGFβ stimulation in at least one of the studies (Additional file 2). Although a majority (2129, 77%) of the genes were identified from one study, which may reflect the diversity of TGFβ transcriptional responses in different tissues and species, core TGFβ-responsive genes were identified in multiple studies showing the independence of tissue and species origins. 445 (17%) genes were identified in 2 independent studies and 175 (6%) genes were identified in at least 3 independent studies. Representative TGFβ-responsive genes with references are shown in Additional file 1. Gene ontology annotation showed that these genes are involved in a wide variety of biological functions where TGFβ plays a role, such as cell growth control, angiogenesis, signal transduction, immune response, cell adhesion, cell motility, and regulation of transcription.

**Figure 2:**
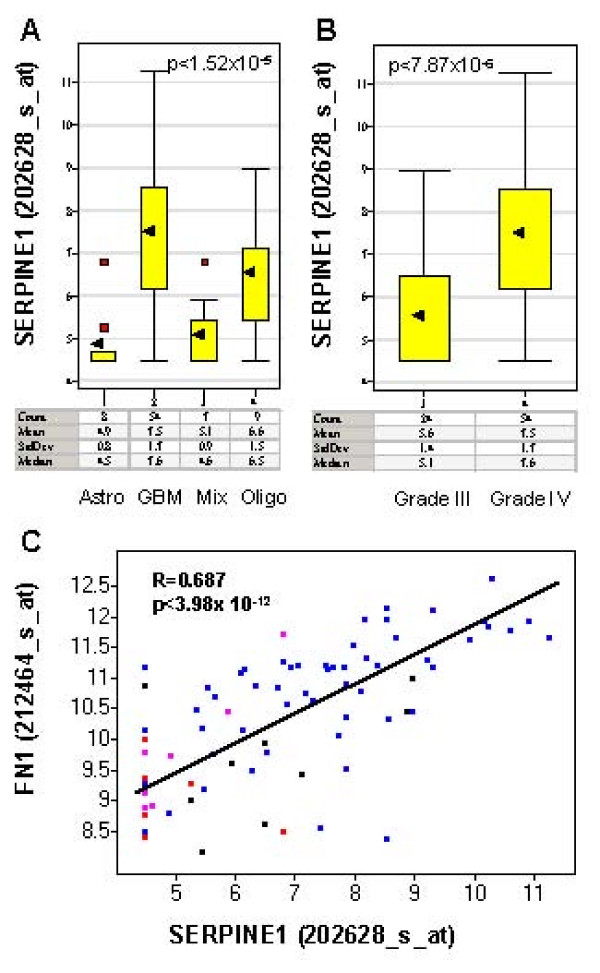
**The expression of TGFβ downstream targets *SERPINE1 *in glial tumors (the Freije dataset) shown in box plots**. Y-axis is the expression level of *SERPINE1 *in log2 scale. The black arrow indicates the mean expression level of *SERPINE1 *in each type of gliomas. Red spots indicate the outlier samples. The table underneath of the box plots are the summary statistics (count, mean, standard deviation (StdDev), median) of the expression level of *SERPINE1 *by glioma types. A: Significant association of *SERPINE1 *expression and histology classification. *SERPINE1 *is significantly upregulated in glioblastoma (GBM) compared to anaplastic astrocytoma (Astro), anaplastic oligodendroglioma (Oligo) and mixed glioma, anaplastic oligoastrocytoma (Mix). The mean expression level of *SERPINE1 *is 6.1-fold higher in glioblastoma compared to anstrocytoma, 5.3-fold higher compared to mixed glioma and 1.9-folder higher compared to oligodendroglioma. P-value computed using ANOVA is indicated at the top right corner of the plot. B. Significant association of *SERPINE1 *expression and the grade of the tumor. *SERPINE1 *is significantly upregulated in grade IV tumors (GBM) compared to grade III tumors (Astro, Oligo, Mix). The mean expression level of *SERPINE1 *is 3.7-fold higher in grade IV tumors (GBM) than in grade III tumors. The P-value was computed using a t-test as indicated in the top left corner of the plot. C. The expression of *SERPINE1 *is highly correlated with *FN1 *expression in gliomas. The correlation coefficient (R) and P-value of correlation (p) were indicated in the plot. The histology types of the gliomas are indicated by colors (blue: GBM, red: Astro, pink: Mix, black: Oligo).

As a first step towards characterizing the TGFβ-responsive gene set in gliomas, we examined the expression of a classic TGFβ target gene *SERPINE1 *in glial tumors within the Freije data set. The expression of *SERPINE1*, also called *PAI-1*, has been shown to be regulated by TGFβ in several reports [31]. Multiple TGFβ-responsive elements have been identified at the promoter region of the *SERPINE1 *gene [32,33]. The protein products of the *SERPINE1 *gene play important roles in TGFβ-mediated biological processes such as fibrosis and wound healing [34]. The induction of *SERPINE1 *expression by TGFβ was abolished by agents that interfered with TGFβ signaling [17]. Our ANOVA analysis of the Freije study suggested that there was no significant association between *SERPINE1 *expression and age or gender. However, *SERPINE1 *expression was significantly associated with the following histological types: glioblastoma (GBM), anaplastic astrocytoma (Astro), anaplastic oligodendroglioma (Oligo) and mixed glioma, anaplastic oligoastrocytoma (Mix)(p < 1.52 × 10^-5^), as well as grades (III and IV) (p < 7.87 × 10^-6^). *SERPINE1 *expression was significantly upregulated in glioblastoma (grade IV) compared to other grade III glial tumors (anaplastic astrocytoma, anaplastic oligodendroglioma and mixed glioma, anaplastic oligoastrocytoma, Figure 2A and Figure 2B). Similar results were found in another TGFβ target *FN1 *(Additional file 2). Moreover, the expressions of *SERPINE1 *and *FN1 *were highly correlated among the high-grade gliomas (correlation coefficient r = 0.687, Figure 2C), suggesting the activation of TGFβ pathway [35]. We also found similar expression patterns in a second independent glioma dataset, the Nutt study [28].

Similar to *SERPINE1 *and *FN1*, the expression of many other well-known TGFβ downstream targets was significantly upregulated in glioblastoma (grade IV) compared to grade III glial tumors, and they are highly correlated with *SERPINE1 *(Additional File 1), including *TGIF *(p < 1.11 × 10^-8^, r = 0.57), *VEGF *(p < 7.57 × 10^-7 ^r = 0.63), *THBS1 *(p < 0.005, r = 0.80), *TIMP1 *(p < 2.5 × 10^-7^, r = 0.80), *COL4A1 *(p < 1.7 × 10^-7^, r = 0.62), *COL1A2 *(p < 8.88 × 10^-7^, r = 0.69) [20,36-38]. Among the 2749 TGFβ-responsive gene set, 2708 unique genes were represented by 7173 array elements in the Freije study [25]. Among the 7173 probesets representing the TGFβ-responsive genes, 417 representing 323 unique genes were significantly upregulated in glioblastomas compared to grade III gliomas with p < 0.001 and fold change >1.5. 1588 probesets representing 997 unique genes were significantly correlated with SERPINE1 with p < 0.001. The complete TGFβ-responsive gene set is summarized in Additional file 2.

### Assessment of TGFβ activation in gliomas using the TGFβ-Responsive gene set

**Figure 3:**
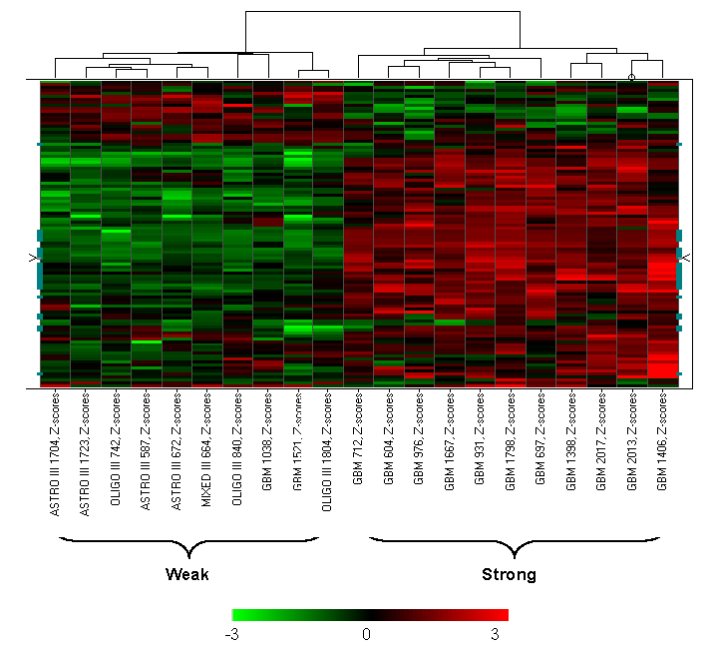
**The SVM training set showing distinct weak or strong TGFβ response pattern in the 103 classifiers that were selected from the most consistent TGFβ-responsive genes (in the Freije dataset)**. The data were Z-score transformed and the color range was indicated by the color bar below the heatmap. Each column represents a tumor sample and the tumor identification number is shown at the bottom of the column. These tumors were selected as training set for the SVM algorithm. Each row represents one of the 103 TGFβ-responsive probesets that were selected from the most consistent TGFβ-responsive genes. The orders of these genes are shown in Additional file 2.

Initially the activation of TGFβ in gliomas was assessed by unsupervised hierarchical clustering of glial tumor microarray data from the Freije study [25] using the most consistent TGFβ-responsive genes in the set (Additional file 1). A TGFβ-responsive classifier set (103 probe sets representing 60 unique genes) was selected as the classifiers using the following criteria: 1) they have been identified to respond to TGFβ stimulation in at least 3 studies; 2) they were consistently up- or down-regulated by TGFβ stimulation in a majority of these studies (absolute direction counts > 50% of total study counts); 3) they varied among all tumors in the Freije dataset (CV >10%) [25]. By visual inspection of the hierarchical clustering results, we identified two small subsets of the glial tumors that showed distinct patterns of the 103 TGFβ-responsive classifiers (Figure 3): one with higher expression of many molecules that were induced by TGFβ *in vitro *and were known as classical TGFβ downstream targets, including *SERPINE1*, *FN1*, *THBS1, COL6A1, COL4A1, COL1A2, LTBP2*, *ITGB5 *(Figure 3, highlighted in green, see Additional file 2 for the order of 103 probe sets), therefore represented strong TGFβ transcriptional response (right, 11 tumors). In contrast, the expression of these molecules was much lower in the other cluster, which represented weak TGFβ transcriptional response (left, 10 tumors). Grade III tumors (8 out of 10) were the majority in the weak TGFβ response cluster, while the strong TGFβ response cluster contained all glioblastomas (Figure 3). The status of TGFβ activation in the remaining tumors is unclear from visual inspection of the hierarchical clustering results.

Support vector machine algorithm was then used to further classifying the TGFβ transcriptional responses among the remaing glial tumors. The 11 tumors in the subset showing strong TGFβ transcriptional responses and the 10 tumors in the weak TGFβ transcriptional responses group (Figure 3) served as the training set. The machine learning was restricted to the 7173 TGFβ-responsive probe sets. The Leave-two-out cross-validation showed 100% accuracy among the training set, suggesting clear distinction between the two subgroups. The rest of the glioma samples were then subjected to SVM as the test set. Table 1 summarized the results of the SVM classification. In total, the majority of the grade III (96%) tumors with one exception were classified as weak TGFβ response group, while over half of grade IV glioblastomas (59%) were classified as strong TGFβ responses, suggesting that TGFβ is more commonly activated in glioblastomas. However, among glioblastomas, the level of TGFβ activation, as assessed by TGFβ-induced transcriptional response, is quite heterogeneous.

**Table 1:** Summary of TGFβ transcriptional responses from SVM Classification of Glial Tumors in the Freije Study and the Nutt Study

	Freije *et al*		Nutt *et al*	
Grade	Weak	Strong	Weak	Strong

Training Set				
III	8	0	6	1
IV	2	11	2	7
Test Set				
III	15	1	12	3
IV	20	21	6	9
Total				
III	23(96%)	1(4%)	18(82%)	4(18%)
IV	22(41%)	32(59%)	8(33%)	16(67%)

**Figure 4:**
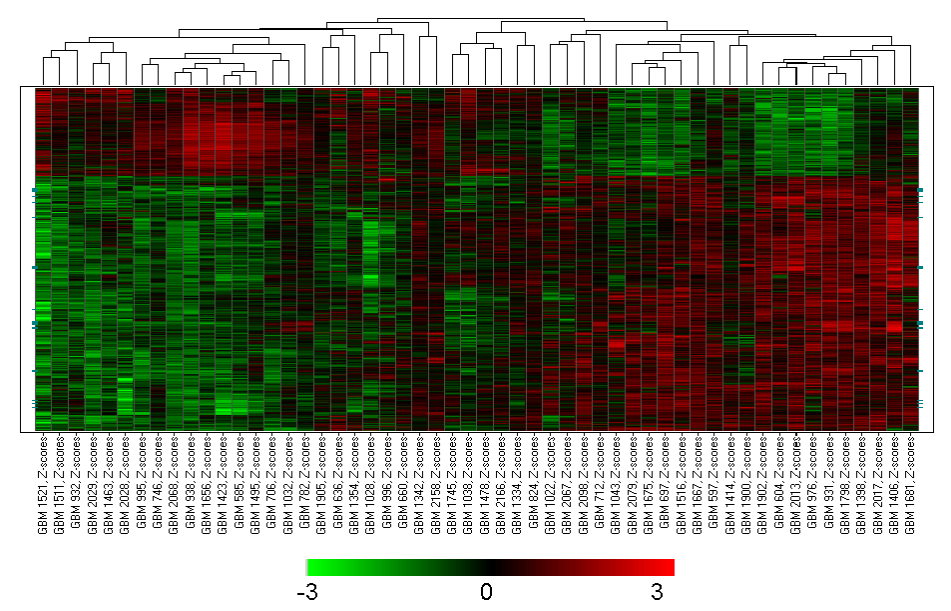
**Differentially expressed genes in the two subgroups of glioblastomas with strong and weak TGFβ response (in the Freije dataset)**. The data were Z-score transformed and the color range was indicated by the color bar below the heatmap. Each column represents a glioblastoma sample and the tumor identification number is shown at the bottom of the column. Each row represents one of the 1386 differentially expressed gene with p < 0.001 and fold change >1.7. The classical TGFβ downstream targets in Table 2 are highlighted as green.

To further examining the differential gene expressions between the two TGFβ response glioblastomas subgroups, we employed the student t test for each gene and the results are shown in Additional file 3. A total of 3497 probesets had a p value of less than 0.001, including 1386 that had a fold change larger than 1.7. This set represented 982 unique known genes and 97 unknown genes, and their differential gene expression patterns among the glioblastomas are shown in Figure 4. P values and mean fold changes for representative TGFβ downstream targets (highlighted in green in Figure 4) are shown in Table 2. The expressions of these TGFβ downstream targets were highly elevated in TGFβ strong response glioblastomas compared to those in TGFβ weak response glioblastoma subgroup, confirming the heterogenenous activation of TGFβ pathway in glioblastomas.

**Table 2:** The Expression of TGFβ downstream targets between the weak and strong TGFβ response groups in Glioblastomas

		**Freijie *et al***		**Nutt *et al***	
**Gene Title**	**Gene Symbol**	**p Value**	**Fold Change**	**p Value**	**Fold Change**

collagen, type I, alpha 1	COL1A1	8.55E-09	6.68	0.018768	2.93
collagen, type I, alpha 2	COL1A2	4.13E-10	4.36	7.10E-05	10.38
collagen, type III, alpha 1 (Ehlers-Danlos syndrome type IV, autosomal dominant)	COL3A1	6.22E-09	5.61	0.002025	5.30
collagen, type IV, alpha 1	COL4A1	7.71E-09	8.38	0.000171	5.48
collagen, type IV, alpha 2	COL4A2	4.75E-09	5.20	4.19E-05	7.69
collagen, type V, alpha 1	COL5A1	4.35E-10	3.82	0.002531	-1.11
collagen, type V, alpha 2	COL5A2	3.52E-09	3.95	5.43E-07	5.14
collagen, type VI, alpha 1	COL6A1	6.40E-07	3.09	2.48E-05	4.95
collagen, type VI, alpha 2	COL6A2	4.04E-11	6.79	4.24E-05	25.45
Collagen, type VIII, alpha 1	COL8A1	1.94E-08	4.52	0.122094	1.27
fibronectin 1	FN1	2.10E-07	2.43	5.45E-05	3.77
serine (or cysteine) proteinase inhibitor, clade E (nexin, plasminogen activator inhibitor type 1), member 1	SERPINE1	1.54E-09	5.69	0.000334	10.83
TGFB-induced factor (TALE family homeobox)	TGIF	1.71E-05	1.83	1.63E-06	3.41
thrombospondin 1	THBS1	2.17E-08	4.28	0.301818	1.29
tissue inhibitor of metalloproteinase 1 (erythroid potentiating activity, collagenase inhibitor)	TIMP1	1.22E-15	6.46	4.19E-06	23.22
vascular endothelial growth factor	VEGF	5.23E-06	3.32	5.72E-06	10.90

TGFβ activation is associated with tumor progression and recurrence. In 4 out of 6 cases where primary and recurrent tumor samples from the same patients were available, TGFβ response in the recurrent glioblastomas became strong from the weak status in the primary tumors (Table 3). No significant survival difference between the two TGFβ response groups in glioblastomas was observed with standard treatments (data not shown), though their potential response to anti-TGFβ therapies may be different.

**Table 3:** Association of TGFβ responses with tumor progression and recurrence.

**Tumor**	**Type**	**TGFb activation class**
MIXED III 886	Primary	Weak
GBM 1463	Recurrent	Weak

OLIGO III 975	Primary	Weak
GBM 1028	Recurrent	Weak

OLIGO III 744	Primary	Weak
GBM 996	Recurrent	Strong

OLIGO III 840	Primary	Weak
GBM 1334	Recurrent	Strong

GBM 938	Primary	Weak
GBM 1406	Recurrent	Strong

GBM 2028	Primary	Weak
GBM 2029	Primary	Weak
GBM 2067	Recurrent	Strong
GBM 2068	Recurrent	Weak

Primary and recurrent tumors from the same patient were grouped together.

### Validation of TGFβ transcriptional response patterns in an independent gliomas microarray study

An independent microarray gene expression dataset containing 28 glioblastoma and 22 anaplastic oligodendroglioma were obtained from Nutt *et al *[28]. The Nutt dataset was generated using the Affymatrix U95A platform that includes about 12000 probe sets. Using the same criteria described above, 101 probe sets representing 72 unique genes were selected from the most consistent TGFβ-responsive genes. 47 of the 72 genes overlap with those used in the Freije study [25]. Subgroups of TGFβ responses similar to those seen in the Freije study [25] were also found by unsupervised clustering (data not shown). SVM classification was used among 3095 probe sets representing TGFβ responsive genes, with a training set of 8 samples showing weak TGFβ response and 8 samples showing strong TGFβ response in the hierarchical clustering analysis. The summary of the TGFβ response subgroups from the Nutt study [28] is also shown in Table 1. Overall, the results from the Nutt dataset were consistent with our results from the Freije dataset [25]. The majority of grade III anaplastic oligodendrogliomas (82%) showed weak TGFβ response while the majority of grade IV glioblastoma (67%) showed strong TGFβ response. Similar to the observations in the Freije study [25], TGFβ activation is heterogeneous. The expressions of many well-known TGFβ downstream targets were significantly different between the two TGFβ response subgroups within glioblastomas (Table 2).

## Discussion

Antagonizing the biological effects of TGFβ has become a potential experimental strategy to treat glioblastoma, one of the most devastating human cancers. Several anti-TGFβ therapies have shown promise in both preclinical and early clinical trials [39]. The current rationale for TGFβ antagonism includes its role in tumor promotion, migration and invasion, metastasis, and tumor-induced immunosuppression. Numerous reports suggest aberrant TGFβ activation in glioblastoma and other high-grade gliomas. This includes abnormal expression of the ligands, more specifically TGFB2 and higher levels of phosphorylated SMADs. However, to date, none of these reports has systematically examined the components of TGFβ signaling to gain a comprehensive view of TGFβ activation in a large cohort of human glioma patients. In this study, we adopted an alternative approach. By examining the transcriptional responses induced by TGFβ activation in publicly available microarray data, we identified two subgroups of glioblastomas that showed distinct patterns of TGFβ activation in two independent studies. Combining the two independent microarray studies of high-grade gliomas, we found that the grade IV glioblastomas showed stronger TGFβ induced transcriptional response than the grade III tumors. In addition, among glioblastomas, 48 out of 78 (62%) showed strong TGFβ activation, while the remaining 38% showed a much weaker TGFβ transcriptional response. How effective the anti-TGFβ therapies would be in the two subgroups of glioblastomas showing distinct TGFβ activation patterns is an open question for future clinical trials. Nevertheless, this study confirmed the previous notion that TGFβ activation occurs commonly in a large portion of glioblastomas, and anti-TGFβ therapies are likely to be beneficial for those patients.

By examining the genes differentially expressed between the two identified subgroups of glioblastomas that showed different TGFβ transcriptional responses, we found that the ligands TGFB1, TGFB2 and their receptors were expressed significantly higher in the strong TGFβ response group (Additional file 3) compared to those in the weak TGFβ response group, suggesting that increased expression of the ligands and receptors contributed to TGFβ activation. THBS1, an activator of TGFβ, was shown to have a higher level in the strong TGFβ response group in one study, suggesting that TGFβ activation may also result from increased bioavailability. In contrast, SMAD7, a negative regulator of TGFβ pathway that often was induced upon TGFβ stimulation *in vitro *(Additional file 1), was downregulated in the strong TGFβ response group (fold change -1.48, p < 0.0007), suggesting the tumor-specific escape of the negative feedback mechanism may also contributed to TGFβ activation in glioblastomas. In addition, genes involved in antigen presentation were upregulated in the TGFβ strong response glioblastomas. These included the genes encoding class I major histocompatibility complex proteins HLA-A, HLA-B, HLA-C, HLA-E, HLA-F, HLA-G, class II major histocompatibility complex proteins HLA-DMA, HLA-DMB, HLA-DPA1, HLA-DPB1, HLA-DQB1, HLA-DRA, HLA-DRB1, MHC class I binding protein CANX, immunoproteosomal subunits PSMB8 and PSMB9, and MHC peptide transport protein TAP1. The upregulation of antigen presentation molecules in the TGFβ strong response glioblastomas suggests that the reported tumor-mediated immunosuppression in glioblastoma occurs through other mechanisms. One study suggested direct targeting of cytotoxic T cell functions by TGFβ and downregulation of the expression of five cytolytic molecules perforin, granzyme A, granzyme B, Fas ligand and interferon γ in T lymphocytes [40]. Strong TGFβ response glioblastomas identified in this study also showed higher expression of many molecules involved in integrin signaling (*ACTA2*, *ACTN1*, *ACTN4*, *ARPC4*, *COL1A1*, *COL1A2*, *COL4A1*, *COL4A2*, *DIRAS3*, *FN1*, *ITGA2*, *ITGA3*, *ITGA4*, *ITGA7*, *ITGB1*, *ITGB2, ITGB4*, *ITGB5*, *LAMA4*, *LAMB1*, *LAMB2*, *LAMC1*, *MRCL3*, *RAP2B*, *RHOC*, *RHOJ*, *RRAS*, *SHC1*, *VASP*, and *ZYX*). Integrins have been shown to mediate the activation of TGFβ [41] and TGFβ is known to regulate the expression of cell adhesion molecules including integrins [42,43]. Interestingly, the glioblastoma group that showed a strong TGFβ response also showed higher expression of the molecules involved in angiogenesis, such as *VEGF*, *FLT1*, *NRP1*, *NRP2*, *ANGPT2*, *JAG1*, *ARTS1*, *TNFRSF12A*. Also the gene expression of a group of insulin-like growth factor binding proteins, including *IGFBP2*, *IGFBP3*, *IGFBP4*, *IGFBP5*, and *IGFBP7 *were significantly higher in TGFβ strong response glioblastomas. Interestingly, *IGFBP2*, one of the most significant gene changes between the two subgroups of glioblastomas showing different TGFβ responses (fold change 7.37, p < 1.27 × 10^-9^), has been shown to enhance glioblastoma invasion [44]. In contrast, the molecules involved in GABA receptor signaling (*GABBR1*, *GABRA1*, *GABRA5*, *GABRB1*, *GABRB3*, *GABRG2*, *GAD1*, *GPR51*) and glutamate receptor signaling (*GLS*, *GRIA2*, *GRIA4*, *GRM1*, *GRM5*, *GRM7*, *SLC17A6*, *SLC17A7*, *SLC1A1*) were downregulated in the TGFβ strong response glial tumors. *BMP2*, a member of TGFβ superfamily that has been shown to promote GABAergic neuron differentiation [45], was also downregulated in the TGFβ strong response glioblastomas (Fold change -2.43, p < 0.0013). These genes differentially expressed between the two identified subgroups of glioblastomas that showed different TGFβ transcriptional responses provide insights into the potential mechanisms of TGFβ-mediated tumor progression and invasion in glioblastomas.

EGFR amplification and PTEN mutations/10q LOH are frequent genetic alterations observed in glioblastomas. Recently a gene signature generated from autocrine platelet-derived growth factor (PDGF) signaling in gliomas has been used to classify gliomas, and it was shown that EGFR amplification and PTEN mutation/10q LOH were largely enriched in the cluster showing weak autocrine PDGF signaling [46]. Using the same signature, we found the TGFβ strong response cluster overlapped with the weak autocrine PGDG signaling subgroup extensively (data not shown), suggesting potential collaboration between EGFR/PTEN/PI-3K pathway and TGFβ pathway in glioblastoma development and progression. Numerous evidence *in vitro *also showed the collaborating roles of EGFR and TGFβ in inducing epithelial to mesenchymal transition, an event that contributes to cell migration, invasion, cell survival and angiogenesis [47-50]. Future studies will be needed to examine if EGFR amplification and PTEN mutation/10q LOH were enriched in the subgroups of glioblastomas that showed strong TGFβ transcriptional response.

## Conclusion

Using the TGFβ-responsive genes we compiled from various studies, we examined the status of TGFβ pathway activation in high-grade gliomas in two independent, publicly available, large-scale gene expression datasets. The purpose of this manuscript is not to establish or test a gene signature that can be used to prospectively classify future datasets in a platform-independent fashion. Rather our goal is to examine the status of TGFβ activation and its heterogeneity among glioblastomas. Therefore, we applied the same methodology/algorithm in two independent datasets and found similar results. Consistent with previous reports, we found that glioblastomas showed a stronger TGFβ response than grade III gliomas. More importantly, among glioblastmas, two subgroups with distinct patterns of TGFβ activation were identified. This molecular stratification of glial tumors using TGFβ transcriptional response is potentially relevant to TGFβ-targeted therapies. A small subset of the gene signatures with classification power are currently under investigation to identify biomarkers that potentially can be used in the clinical setting with anti-TGFβ therapies.

## Competing interests

AMK, while employed by Scios Inc., held stock options in the company.

## Authors' contributions

Both authors have read and approved the final manuscript. XLX conducted the study and prepared the manuscript. AMK supervised the study and edited the manuscript.

## Supplementary Material

Additional File 1 **The representative TGFβ-responsive genes.** Click here for file

Additional File 2 **Complete TGFβ-responsive gene set in glial tumors in the Freije dataset.** Click here for file

Additional File 3 **The difference of gene expression between the two subgroups of glioblastomas showing different TGFβ responses in the Freije dataset.** Click here for file

## References

[B1] Jemal A, Siegel R, Ward E, Murray T, Xu J, Smigal C, Thun MJ (2006). Cancer statistics, 2006. CA: a Cancer Journal for Clinicians.

[B2] Stewart LA (2002). Chemotherapy in adult high-grade glioma: a systematic review and meta-analysis of individual patient data from 12 randomised trials. Lancet.

[B3] Kitange GJ, Templeton KL, Jenkins RB (2003). Recent advances in the molecular genetics of primary gliomas. Current Opinion in Oncology.

[B4] Choe G, Horvath S, Cloughesy TF, Crosby K, Seligson D, Palotie A, Inge L, Smith BL, Sawyers CL, Mischel PS (2003). Analysis of the phosphatidylinositol 3'-kinase signaling pathway in glioblastoma patients in vivo. Cancer Research.

[B5] Rich JN, Reardon DA, Peery T, Dowell JM, Quinn JA, Penne KL, Wikstrand CJ, Van Duyn LB, Dancey JE, McLendon RE (2004). Phase II trial of gefitinib in recurrent glioblastoma. Journal of Clinical Oncology.

[B6] Mellinghoff IK, Wang MY, Vivanco I, Haas-Kogan DA, Zhu S, Dia EQ, Lu KV, Yoshimoto K, Huang JH, Chute DJ (2005). Molecular determinants of the response of glioblastomas to EGFR kinase inhibitors[see comment][erratum appears in N Engl J Med. 2006 Feb 23;354(8):884]. New England Journal of Medicine.

[B7] Samuels V, Barrett JM, Bockman S, Pantazis CG, Allen MB (1989). Immunocytochemical study of transforming growth factor expression in benign and malignant gliomas. American Journal of Pathology.

[B8] Kjellman C, Olofsson SP, Hansson O, Von Schantz T, Lindvall M, Nilsson I, Salford LG, Sjogren HO, Widegren B (2000). Expression of TGF-beta isoforms, TGF-beta receptors, and SMAD molecules at different stages of human glioma. International Journal of Cancer.

[B9] Horst HA, Scheithauer BW, Kelly PJ, Kovach JS (1992). Distribution of transforming growth factor-beta 1 in human astrocytomas. Human Pathology.

[B10] Yamada N, Kato M, Yamashita H, Nister M, Miyazono K, Heldin CH, Funa K (1995). Enhanced expression of transforming growth factor-beta and its type-I and type-II receptors in human glioblastoma. International Journal of Cancer.

[B11] Weller M, Fontana A (1995). The failure of current immunotherapy for malignant glioma. Tumor-derived TGF-beta, T-cell apoptosis, and the immune privilege of the brain. Brain Research – Brain Research Reviews.

[B12] Reiss M (1997). Transforming growth factor-beta and cancer: a love-hate relationship?. Oncology Research.

[B13] Cui W, Fowlis DJ, Bryson S, Duffie E, Ireland H, Balmain A, Akhurst RJ (1996). TGFbeta1 inhibits the formation of benign skin tumors, but enhances progression to invasive spindle carcinomas in transgenic mice. Cell.

[B14] Oft M, Peli J, Rudaz C, Schwarz H, Beug H, Reichmann E (1996). TGF-beta1 and Ha-Ras collaborate in modulating the phenotypic plasticity and invasiveness of epithelial tumor cells. Genes & Development.

[B15] Yin JJ, Selander K, Chirgwin JM, Dallas M, Grubbs BG, Wieser R, Massague J, Mundy GR, Guise TA (1999). TGF-beta signaling blockade inhibits PTHrP secretion by breast cancer cells and bone metastases development. Journal of Clinical Investigation.

[B16] Feng XH, Derynck R (2005). Specificity and versatility in tgf-beta signaling through Smads. Annual Review of Cell & Developmental Biology.

[B17] Kapoun AM, Gaspar NJ, Wang Y, Damm D, Liu YW, O'Young G, Quon D, Lam A, Munson K, Tran TT (2006). Transforming growth factor-beta receptor type 1 (TGFbetaRI) kinase activity but not p38 activation is required for TGFbetaRI-induced myofibroblast differentiation and profibrotic gene expression. Mol Pharmacol.

[B18] Kapoun AM, Liang F, O'Young G, Damm DL, Quon D, White RT, Munson K, Lam A, Schreiner GF, Protter AA (2004). B-type natriuretic peptide exerts broad functional opposition to transforming growth factor-beta in primary human cardiac fibroblasts: fibrosis, myofibroblast conversion, proliferation, and inflammation. Circ Res.

[B19] Valcourt U, Kowanetz M, Niimi H, Heldin CH, Moustakas A (2005). TGF-beta and the Smad signaling pathway support transcriptomic reprogramming during epithelial-mesenchymal cell transition. Molecular Biology of the Cell.

[B20] Verrecchia F, Chu ML, Mauviel A (2001). Identification of novel TGF-beta/Smad gene targets in dermal fibroblasts using a combined cDNA microarray/promoter transactivation approach. Journal of Biological Chemistry.

[B21] Xie L, Law BK, Aakre ME, Edgerton M, Shyr Y, Bhowmick NA, Moses HL (2003). Transforming growth factor beta-regulated gene expression in a mouse mammary gland epithelial cell line. Breast Cancer Research.

[B22] Zavadil J, Bitzer M, Liang D, Yang YC, Massimi A, Kneitz S, Piek E, Bottinger EP (2001). Genetic programs of epithelial cell plasticity directed by transforming growth factor-beta. Proceedings of the National Academy of Sciences of the United States of America.

[B23] Yang YC, Piek E, Zavadil J, Liang D, Xie D, Heyer J, Pavlidis P, Kucherlapati R, Roberts AB, Bottinger EP (2003). Hierarchical model of gene regulation by transforming growth factor beta. Proceedings of the National Academy of Sciences of the United States of America.

[B24] Levy L, Hill CS (2005). Smad4 dependency defines two classes of transforming growth factor {beta} (TGF-{beta}) target genes and distinguishes TGF-{beta}-induced epithelial-mesenchymal transition from its antiproliferative and migratory responses. Molecular & Cellular Biology.

[B25] Freije WA, Castro-Vargas FE, Fang Z, Horvath S, Cloughesy T, Liau LM, Mischel PS, Nelson SF (2004). Gene expression profiling of gliomas strongly predicts survival. Cancer Research.

[B26] Rich JN, Hans C, Jones B, Iversen ES, McLendon RE, Rasheed BK, Dobra A, Dressman HK, Bigner DD, Nevins JR, West M (2005). Gene expression profiling and genetic markers in glioblastoma survival. Cancer Research.

[B27] Liang Y, Diehn M, Watson N, Bollen AW, Aldape KD, Nicholas MK, Lamborn KR, Berger MS, Botstein D, Brown PO, Israel MA (2005). Gene expression profiling reveals molecularly and clinically distinct subtypes of glioblastoma multiforme. Proceedings of the National Academy of Sciences of the United States of America.

[B28] Nutt CL, Mani DR, Betensky RA, Tamayo P, Cairncross JG, Ladd C, Pohl U, Hartmann C, McLaughlin ME, Batchelor TT (2003). Gene expression-based classification of malignant gliomas correlates better with survival than histological classification. Cancer Research.

[B29] Schadt EE, Li C, Ellis B, Wong WH (2001). Feature extraction and normalization algorithms for high-density oligonucleotide gene expression array data. J Cell Biochem Suppl.

[B30] Gaspar NJ, Li L, Kapoun AM, Medicherla S, Reddy M, Li G, O'Young G, Quon D, Henson M, Damm DL (2007). Inhibition of transforming growth factor beta signaling reduces pancreatic adenocarcinoma growth and invasiveness. Mol Pharmacol.

[B31] Gerwin BI, Keski-Oja J, Seddon M, Lechner JF, Harris CC (1990). TGF-beta 1 modulation of urokinase and PAI-1 expression in human bronchial epithelial cells. Am J Physiol.

[B32] Keeton MR, Curriden SA, van Zonneveld AJ, Loskutoff DJ (1991). Identification of regulatory sequences in the type 1 plasminogen activator inhibitor gene responsive to transforming growth factor beta. J Biol Chem.

[B33] Dennler S, Itoh S, Vivien D, ten Dijke P, Huet S, Gauthier JM (1998). Direct binding of Smad3 and Smad4 to critical TGF beta-inducible elements in the promoter of human plasminogen activator inhibitor-type 1 gene. EMBO Journal.

[B34] Dellas C, Loskutoff DJ (2005). Historical analysis of PAI-1 from its discovery to its potential role in cell motility and disease. Thromb Haemost.

[B35] Gold LI (1999). The role for transforming growth factor-beta (TGF-beta) in human cancer. Critical Reviews in Oncogenesis.

[B36] Chen F, Ogawa K, Nagarajan RP, Zhang M, Kuang C, Chen Y (2003). Regulation of TG-interacting factor by transforming growth factor-beta. Biochemical Journal.

[B37] Hocevar BA, Brown TL, Howe PH (1999). TGF-beta induces fibronectin synthesis through a c-Jun N-terminal kinase-dependent, Smad4-independent pathway. EMBO Journal.

[B38] Horiguchi H, Jin L, Ruebel KH, Scheithauer BW, Lloyd RV (2004). Regulation of VEGF-A, VEGFR-I, thrombospondin-1, -2, and -3 expression in a human pituitary cell line (HP75) by TGFbeta1, bFGF, and EGF. Endocrine.

[B39] Akhurst RJ (2006). Large- and small-molecule inhibitors of transforming growth factor-beta signaling. Current Opinion in Investigational Drugs.

[B40] Thomas DA, Massague J (2005). TGF-beta directly targets cytotoxic T cell functions during tumor evasion of immune surveillance[see comment]. Cancer Cell.

[B41] Munger JS, Harpel JG, Giancotti FG, Rifkin DB (1998). Interactions between growth factors and integrins: latent forms of transforming growth factor-beta are ligands for the integrin alphavbeta1. Molecular Biology of the Cell.

[B42] Paulus W, Baur I, Huettner C, Schmausser B, Roggendorf W, Schlingensiepen KH, Brysch W (1995). Effects of transforming growth factor-beta 1 on collagen synthesis, integrin expression, adhesion and invasion of glioma cells. Journal of Neuropathology & Experimental Neurology.

[B43] Lai CF, Feng X, Nishimura R, Teitelbaum SL, Avioli LV, Ross FP, Cheng SL (2000). Transforming growth factor-beta up-regulates the beta 5 integrin subunit expression via Sp1 and Smad signaling. Journal of Biological Chemistry.

[B44] Wang H, Wang H, Shen W, Huang H, Hu L, Ramdas L, Zhou YH, Liao WS, Fuller GN, Zhang W (2003). Insulin-like growth factor binding protein 2 enhances glioblastoma invasion by activating invasion-enhancing genes. Cancer Research.

[B45] Hattori A, Katayama M, Iwasaki S, Ishii K, Tsujimoto M, Kohno M (1999). Bone morphogenetic protein-2 promotes survival and differentiation of striatal GABAergic neurons in the absence of glial cell proliferation. Journal of Neurochemistry.

[B46] Ma D, Nutt CL, Shanehsaz P, Peng X, Louis DN, Kaetzel DM (2005). Autocrine platelet-derived growth factor-dependent gene expression in glioblastoma cells is mediated largely by activation of the transcription factor sterol regulatory element binding protein and is associated with altered genotype and patient survival in human brain tumors. Cancer Research.

[B47] Docherty NG, O'Sullivan OE, Healy DA, Murphy M, O'Neill AJ, Fitzpatrick JM, Watson RW (2006). TGF-beta1-induced EMT can occur independently of its proapoptotic effects and is aided by EGF receptor activation. American Journal of Physiology – Renal Physiology.

[B48] Seton-Rogers SE, Lu Y, Hines LM, Koundinya M, LaBaer J, Muthuswamy SK, Brugge JS (2004). Cooperation of the ErbB2 receptor and transforming growth factor beta in induction of migration and invasion in mammary epithelial cells. Proceedings of the National Academy of Sciences of the United States of America.

[B49] Prevot V, Cornea A, Mungenast A, Smiley G, Ojeda SR (2003). Activation of erbB-1 signaling in tanycytes of the median eminence stimulates transforming growth factor beta1 release via prostaglandin E2 production and induces cell plasticity. Journal of Neuroscience.

[B50] Held-Feindt J, Lutjohann B, Ungefroren H, Mehdorn HM, Mentlein R (2003). Interaction of transforming growth factor-beta (TGF-beta) and epidermal growth factor (EGF) in human glioma cells. Journal of Neuro-Oncology.

