# Supplementary material for: Heterogeneous activation of the TGFβ pathway in glioblastomas identified by gene expression-based classification using TGFβ-responsive genes
Source: J Transl Med. 2009 Feb 3;7:12. doi: 10.1186/1479-5876-7-12 (PMC2655274; doi:10.1186/1479-5876-7-12)
Supplement: Additional File 1 — The representative TGFβ-responsive genes. [file 1479-5876-7-12-S1.doc]

## Additional file 1 - The Representative TGFβ-Responsive Genes

| Symbol | Gene Name | Entrez Gene ID | Counts of Studies* | Directions* | Reference |
| --- | --- | --- | --- | --- | --- |
| ITGB5 | integrin, beta 5 | 3693 | 7 | 6 | [1-6], unpublished result |
| FN1 | fibronectin 1 | 2335 | 6 | 5 | [1-4, 7, 8] |
| SERPINE1 | serine (or cysteine) proteinase inhibitor, clade E (nexin, plasminogen activator inhibitor type 1), member 1 | 5054 | 6 | 5 | [1, 4, 5, 7, 9, 10] |
| CMKOR1 | chemokine orphan receptor 1 | 57007 | 6 | 5 | [[3, 4, 7, 9, 11], unpublished result |
| LTBP2 | latent transforming growth factor beta binding protein 2 | 4053 | 6 | 5 | [4, 5, 7, 9, 12], unpublished result |
| MYC | v-myc myelocytomatosis viral oncogene homolog (avian) | 4609 | 5 | -4 | [1-3, 7, 13] |
| IFIT1 | interferon-induced protein with tetratricopeptide repeats 1 | 3434 | 5 | -4 | [4, 5, 7, 11] unpublished result |
| FHL2 | four and a half LIM domains 2 | 2274 | 5 | 5 | [2-4, 7, 9] |
| PDGFA | platelet-derived growth factor alpha polypeptide | 5154 | 5 | 4 | [2, 3, 5, 7, 14] |
| SMAD7 | SMAD, mothers against DPP homolog 7 (Drosophila) | 4092 | 5 | 4 | [5, 7, 9, 15], unpublished result |
| ZYX | zyxin | 7791 | 4 | 4 | [1-4] |
| LAMB1 | laminin, beta 1 | 3912 | 4 | 3 | [1, 2, 4, 16] |
| ID3 | inhibitor of DNA binding 3, dominant negative helix-loop-helix protein | 3399 | 4 | -4 | [2-4, 9] |
| PDGFB | platelet-derived growth factor beta polypeptide (simian sarcoma viral (v-sis) oncogene homolog) | 5155 | 4 | 3 | [4, 5, 14], unpublished result |
| COL4A1 | collagen, type IV, alpha 1 | 1282 | 4 | 3 | [2, 4, 9, 16] |
| SLC29A1 | solute carrier family 29 (nucleoside transporters), member 1 | 2030 | 4 | 4 | [2, 4, 5], unpublished result |
| ITGB1 | integrin, beta 1 (fibronectin receptor, beta polypeptide, antigen CD29 includes MDF2, MSK12) | 3688 | 4 | 3 | [2, 4, 7, 17] |
| IL1RAP | interleukin 1 receptor accessory protein | 3556 | 4 | 4 | [4, 5, 7], unpublished data |
| IER3 | immediate early response 3 | 8870 | 4 | 4 | [2, 5, 7, 9] |
| CALD1 | caldesmon 1 | 800 | 4 | 4 | [2, 3, 5], unpublished data |
| ACTN1 | actinin, alpha 1 | 87 | 4 | 4 | [2, 5, 7, 9] |
| CD59 | CD59 antigen p18-20 (antigen identified by monoclonal antibodies 16.3A5, EJ16, EJ30, EL32 and G344) | 966 | 3 | 2 | [1, 7, 18] |
| ITGB2 | integrin, beta 2 (antigen CD18 (p95), lymphocyte function-associated antigen 1; macrophage antigen 1 (mac-1) beta subunit) | 3689 | 3 | 2 | [1, 4, 19] |
| ITGA3 | integrin, alpha 3 (antigen CD49C, alpha 3 subunit of VLA-3 receptor) | 3675 | 3 | 2 | [1, 2, 20] |
| COL6A1 | collagen, type VI, alpha 1 | 1291 | 3 | 2 | [1, 3, 21] |
| COL1A2 | collagen, type I, alpha 2 | 1278 | 3 | 2 | [1, 7, 22] |
| CSPG2 | chondroitin sulfate proteoglycan 2 (versican) | 1462 | 3 | 2 | [1, 7, 23] |
| MMP1 | matrix metalloproteinase 1 (interstitial collagenase) | 4312 | 3 | 2 | [1, 4, 16] |
| TNC | tenascin C (hexabrachion) | 3371 | 3 | 2 | [1, 4, 24] |
| THBS1 | thrombospondin 1 | 7057 | 3 | 2 | [1, 4, 25] |
| ALDH3B1 | aldehyde dehydrogenase 3 family, member B1 | 221 | 3 | -3 | [4, 7], unpublished data |
| BMP1 | bone morphogenetic protein 1 | 649 | 3 | 3 | [2-4] |
| FOS | v-fos FBJ murine osteosarcoma viral oncogene homolog | 2353 | 3 | 2 | [3, 4, 26] |
| FOSB | FBJ murine osteosarcoma viral oncogene homolog B | 2354 | 3 | 2 | [4, 11], unpublished data |
| DUSP1 | dual specificity phosphatase 1 | 1843 | 3 | 2 | [4, 27, 28] |
| CDKN1A | cyclin-dependent kinase inhibitor 1A (p21, Cip1) | 1026 | 3 | 2 | [4, 29] |
| NET1 | neuroepithelial cell transforming gene 1 | 10276 | 3 | 2 | [4, 9, 30] |
| B4GALT1 | UDP-Gal:betaGlcNAc beta 1,4- galactosyltransferase, polypeptide 1 | 2683 | 3 | 3 | [2, 4], unpublished data |
| CDKN2B | cyclin-dependent kinase inhibitor 2B (p15, inhibits CDK4) | 1030 | 3 | 2 | [4, 9, 31] |
| PTPRB | protein tyrosine phosphatase, receptor type, B | 5787 | 3 | 3 | [4, 9], unpublished data |
| DLC1 | deleted in liver cancer 1 | 10395 | 3 | 3 | [4, 7, 9] |
| BMPR2 | bone morphogenetic protein receptor, type II (serine/threonine kinase) | 659 | 3 | 3 | [4, 5, 7] |
| PDGFRA | platelet-derived growth factor receptor, alpha polypeptide | 5156 | 3 | -2 | [7, 27, 32] |
| GADD45B | growth arrest and DNA-damage-inducible, beta | 4616 | 3 | 2 | [9, 27, 33] |
| TMEPAI | transmembrane, prostate androgen induced RNA | 56937 | 3 | 3 | [2, 3, 9] |
| SKIL | SKI-like | 6498 | 3 | 3 | [2, 7, 9] |
| GPR56 | G protein-coupled receptor 56 | 9289 | 3 | 3 | [2, 3, 5] |
| RDH10 | retinol dehydrogenase 10 (all-trans) | 157506 | 3 | 3 | [2, 3, 9] |
| EXT1 | exostoses (multiple) 1 | 2131 | 3 | 3 | [2, 3, 7] |
| PPL | periplakin | 5493 | 3 | -3 | [3, 5], unpublished data |
| F3 | coagulation factor III (thromboplastin, tissue factor) | 2152 | 3 | 3 | [3, 7], unpublished data |
| TGM2 | transglutaminase 2 (C polypeptide, protein-glutamine-gamma-glutamyltransferase) | 7052 | 3 | 2 | [3, 5, 34] |
| TGFB2 | transforming growth factor, beta 2 | 7042 | 3 | 3 | [7, 9], unpublished data |
| LUM | lumican | 4060 | 3 | -2 | [7, 35], unpublished data |
| NCF4 | neutrophil cytosolic factor 4, 40kDa | 4689 | 3 | 2 | [7, 11], unpublished data |
| ENPP2 | ectonucleotide pyrophosphatase/phosphodiesterase 2 (autotaxin) | 5168 | 3 | -2 | [7, 36], unpublished data |
| SCD | stearoyl-CoA desaturase (delta-9-desaturase) | 6319 | 3 | 2 | [5, 7, 37] |
| SPOCK | sparc/osteonectin, cwcv and kazal-like domains proteoglycan (testican) | 6695 | 3 | 2 | [5, 38], unpublished data |
| SERPINE2 | serine (or cysteine) proteinase inhibitor, clade E (nexin, plasminogen activator inhibitor type 1), member 2 | 5270 | 3 | 3 | [5, 7], unpublished data |
| SNF1LK | SNF1-like kinase | 150094 | 3 | 3 | [5, 7], unpublished data |

Reference:

1. Verrecchia F, Chu ML, Mauviel A: **Identification of novel TGF-beta /Smad gene targets in dermal fibroblasts using a combined cDNA microarray/promoter transactivation approach.** *Journal of Biological Chemistry* 2001, **276:**17058-17062.

2. Xie L, Law BK, Aakre ME, Edgerton M, Shyr Y, Bhowmick NA, Moses HL: **Transforming growth factor beta-regulated gene expression in a mouse mammary gland epithelial cell line.** *Breast Cancer Research* 2003, **5:**R187-198.

3. Valcourt U, Kowanetz M, Niimi H, Heldin CH, Moustakas A: **TGF-beta and the Smad signaling pathway support transcriptomic reprogramming during epithelial-mesenchymal cell transition.** *Molecular Biology of the Cell* 2005, **16:**1987-2002.

4. Zavadil J, Bitzer M, Liang D, Yang YC, Massimi A, Kneitz S, Piek E, Bottinger EP: **Genetic programs of epithelial cell plasticity directed by transforming growth factor-beta.** *Proceedings of the National Academy of Sciences of the United States of America* 2001, **98:**6686-6691.

5. Gaspar NJ, Li L, Kapoun AM, Medicherla S, Reddy M, Li G, O'Young G, Quon D, Henson M, Damm DL, et al: **Inhibition of transforming growth factor beta signaling reduces pancreatic adenocarcinoma growth and invasiveness.** *Mol Pharmacol* 2007, **72:**152-161.

6. Lai CF, Feng X, Nishimura R, Teitelbaum SL, Avioli LV, Ross FP, Cheng SL: **Transforming growth factor-beta up-regulates the beta 5 integrin subunit expression via Sp1 and Smad signaling.** *Journal of Biological Chemistry* 2000, **275:**36400-36406.

7. Kapoun AM, Gaspar NJ, Wang Y, Damm D, Liu YW, O'Young G, Quon D, Lam A, Munson K, Tran TT, et al: **Transforming growth factor-beta receptor type 1 (TGFbetaRI) kinase activity but not p38 activation is required for TGFbetaRI-induced myofibroblast differentiation and profibrotic gene expression.** *Mol Pharmacol* 2006, **70:**518-531.

8. Hocevar BA, Brown TL, Howe PH: **TGF-beta induces fibronectin synthesis through a c-Jun N-terminal kinase-dependent, Smad4-independent pathway.** *EMBO Journal* 1999, **18:**1345-1356.

9. Levy L, Hill CS: **Smad4 dependency defines two classes of transforming growth factor {beta} (TGF-{beta}) target genes and distinguishes TGF-{beta}-induced epithelial-mesenchymal transition from its antiproliferative and migratory responses.** *Molecular & Cellular Biology* 2005, **25:**8108-8125.

10. Keeton MR, Curriden SA, van Zonneveld AJ, Loskutoff DJ: **Identification of regulatory sequences in the type 1 plasminogen activator inhibitor gene responsive to transforming growth factor beta.** *J Biol Chem* 1991, **266:**23048-23052.

11. Kowanetz M, Valcourt U, Bergstrom R, Heldin CH, Moustakas A: **Id2 and Id3 define the potency of cell proliferation and differentiation responses to transforming growth factor beta and bone morphogenetic protein.** *Mol Cell Biol* 2004, **24:**4241-4254.

12. Ahmed W, Kucich U, Abrams W, Bashir M, Rosenbloom J, Segade F, Mecham R, Rosenbloom J: **Signaling pathway by which TGF-beta1 increases expression of latent TGF-beta binding protein-2 at the transcriptional level.** *Connective Tissue Research* 1998, **37:**263-276.

13. Chen CR, Kang Y, Siegel PM, Massague J: **E2F4/5 and p107 as Smad cofactors linking the TGFbeta receptor to c-myc repression.** *Cell* 2002, **110:**19-32.

14. Abboud SL: **Regulation of platelet-derived growth factor A and B chain gene expression in bone marrow stromal cells.** *Journal of Cellular Physiology* 1995, **164:**434-440.

15. Nakao A, Afrakhte M, Moren A, Nakayama T, Christian JL, Heuchel R, Itoh S, Kawabata M, Heldin NE, Heldin CH, ten Dijke P: **Identification of Smad7, a TGFbeta-inducible antagonist of TGF-beta signalling.[see comment].** *Nature* 1997, **389:**631-635.

16. Douthwaite JA, Johnson TS, Haylor JL, Watson P, El Nahas AM: **Effects of transforming growth factor-beta1 on renal extracellular matrix components and their regulating proteins.** *Journal of the American Society of Nephrology* 1999, **10:**2109-2119.

17. Nejjari M, Hafdi Z, Dumortier J, Bringuier AF, Feldmann G, Scoazec JY: **alpha6beta1 integrin expression in hepatocarcinoma cells: regulation and role in cell adhesion and migration.** *Int J Cancer* 1999, **83:**518-525.

18. Pasch MC, Bos JD, Daha MR, Asghar SS: **Transforming growth factor-beta isoforms regulate the surface expression of membrane cofactor protein (CD46) and CD59 on human keratinocytes [corrected].** *Eur J Immunol* 1999, **29:**100-108.

19. Bauvois B, Van Weyenbergh J, Rouillard D, Wietzerbin J: **TGF-beta 1-stimulated adhesion of human mononuclear phagocytes to fibronectin and laminin is abolished by IFN-gamma: dependence on alpha 5 beta 1 and beta 2 integrins.** *Exp Cell Res* 1996, **222:**209-217.

20. Katabami K, Mizuno H, Sano R, Saito Y, Ogura M, Itoh S, Tsuji T: **Transforming growth factor-beta1 upregulates transcription of alpha3 integrin gene in hepatocellular carcinoma cells via Ets-transcription factor-binding motif in the promoter region.** *Clin Exp Metastasis* 2005, **22:**539-548.

21. Berking C, Takemoto R, Schaider H, Showe L, Satyamoorthy K, Robbins P, Herlyn M: **Transforming growth factor-beta1 increases survival of human melanoma through stroma remodeling.** *Cancer Res* 2001, **61:**8306-8316.

22. Zhang W, Ou J, Inagaki Y, Greenwel P, Ramirez F: **Synergistic cooperation between Sp1 and Smad3/Smad4 mediates transforming growth factor beta1 stimulation of alpha 2(I)-collagen (COL1A2) transcription.** *Journal of Biological Chemistry* 2000, **275:**39237-39245.

23. Venkatesan N, Roughley PJ, Ludwig MS: **Proteoglycan expression in bleomycin lung fibroblasts: role of transforming growth factor-beta(1) and interferon-gamma.** *Am J Physiol Lung Cell Mol Physiol* 2002, **283:**L806-814.

24. Hau P, Kunz-Schughart LA, Rummele P, Arslan F, Dorfelt A, Koch H, Lohmeier A, Hirschmann B, Muller A, Bogdahn U, Bosserhoff AK: **Tenascin-C protein is induced by transforming growth factor-beta1 but does not correlate with time to tumor progression in high-grade gliomas.** *J Neurooncol* 2006, **77:**1-7.

25. McGillicuddy FC, O'Toole D, Hickey JA, Gallagher WM, Dawson KA, Keenan AK: **TGF-beta1-induced thrombospondin-1 expression through the p38 MAPK pathway is abolished by fluvastatin in human coronary artery smooth muscle cells.** *Vascul Pharmacol* 2006, **44:**469-475.

26. Osaki M, Tsukazaki T, Yonekura A, Miyazaki Y, Iwasaki K, Shindo H, Yamashita S: **Regulation of c-fos gene induction and mitogenic effect of transforming growth factor-beta1 in rat articular chondrocyte.** *Endocr J* 1999, **46:**253-261.

27. Yang YC, Piek E, Zavadil J, Liang D, Xie D, Heyer J, Pavlidis P, Kucherlapati R, Roberts AB, Bottinger EP: **Hierarchical model of gene regulation by transforming growth factor beta.** *Proceedings of the National Academy of Sciences of the United States of America* 2003, **100:**10269-10274.

28. Tong XK, Hamel E: **Transforming growth factor-beta 1 impairs endothelin-1-mediated contraction of brain vessels by inducing mitogen-activated protein (MAP) kinase phosphatase-1 and inhibiting p38 MAP kinase.** *Mol Pharmacol* 2007, **72:**1476-1483.

29. Datto MB, Li Y, Panus JF, Howe DJ, Xiong Y, Wang XF: **Transforming growth factor beta induces the cyclin-dependent kinase inhibitor p21 through a p53-independent mechanism.** *Proc Natl Acad Sci U S A* 1995, **92:**5545-5549.

30. Shen X, Li J, Hu PP, Waddell D, Zhang J, Wang XF: **The activity of guanine exchange factor NET1 is essential for transforming growth factor-beta-mediated stress fiber formation.** *J Biol Chem* 2001, **276:**15362-15368.

31. Seoane J, Pouponnot C, Staller P, Schader M, Eilers M, Massague J: **TGFbeta influences Myc, Miz-1 and Smad to control the CDK inhibitor p15INK4b.** *Nat Cell Biol* 2001, **3:**400-408.

32. Yang D, Jin D, Chen J, Jing Z, Wu D: **Modulation of transforming growth factor beta to platelet-derived growth factor receptor-alpha of human osteoblasts.** *Chin Med J (Engl)* 2000, **113:**621-624.

33. Major MB, Jones DA: **Identification of a gadd45beta 3' enhancer that mediates SMAD3- and SMAD4-dependent transcriptional induction by transforming growth factor beta.** *J Biol Chem* 2004, **279:**5278-5287.

34. Fuchshofer R, Birke M, Welge-Lussen U, Kook D, Lutjen-Drecoll E: **Transforming growth factor-beta 2 modulated extracellular matrix component expression in cultured human optic nerve head astrocytes.** *Invest Ophthalmol Vis Sci* 2005, **46:**568-578.

35. Funderburgh JL, Funderburgh ML, Mann MM, Corpuz L, Roth MR: **Proteoglycan expression during transforming growth factor beta -induced keratocyte-myofibroblast transdifferentiation.** *J Biol Chem* 2001, **276:**44173-44178.

36. Kehlen A, Englert N, Seifert A, Klonisch T, Dralle H, Langner J, Hoang-Vu C: **Expression, regulation and function of autotaxin in thyroid carcinomas.** *Int J Cancer* 2004, **109:**833-838.

37. Samuel W, Nagineni CN, Kutty RK, Parks WT, Gordon JS, Prouty SM, Hooks JJ, Wiggert B: **Transforming growth factor-beta regulates stearoyl coenzyme A desaturase expression through a Smad signaling pathway.** *J Biol Chem* 2002, **277:**59-66.

38. Fujita T, Shiba H, Van Dyke TE, Kurihara H: **Differential effects of growth factors and cytokines on the synthesis of SPARC, DNA, fibronectin and alkaline phosphatase activity in human periodontal ligament cells.** *Cell Biol Int* 2004, **28:**281-286.
